# T Follicular Helper Cell Immune Signatures Associated With Disease Severity in Severe Fever With Thrombocytopenia Syndrome

**DOI:** 10.1155/jimr/8984077

**Published:** 2026-06-23

**Authors:** Danning Xu, Ting Wang, Wei Wei, Yun Wang, Rujia Chen, Renren Ouyang, Shiji Wu, Feng Wang, Hongyan Hou

**Affiliations:** ^1^ Department of Laboratory Medicine, Tongji Hospital, Tongji Medical College, Huazhong University of Science and Technology, Jiefang Road 1095, Wuhan, 430030, Hubei Province, China, hust.edu.cn

**Keywords:** severe fever with thrombocytopenia syndrome, T follicular helper (Tfh), Tfh subsets

## Abstract

**Background:**

Severe fever with thrombocytopenia syndrome (SFTS) is marked by high case fatality and profound antiviral immune dysregulation, yet the clinical implications of changes in circulating T follicular helper (Tfh) cells remain unclear.

**Methods:**

We enrolled 79 patients with RT‐PCR‐confirmed SFTSV infection who were hospitalized from May to October 2023 and categorized them as acute‐phase survivors (AS) or acute‐phase deceased (AD). Frequencies of circulating Tfh subsets (Tfh1, Tfh2, Tfh17, and PD‐1^+^ Tfh) were measured by flow cytometry, and plasma SFTSV RNA was quantified by RT‐qPCR. Their relationships with clinical variables and prognosis were analyzed using correlation analysis, ROC analysis, Kaplan–Meier survival analysis, and forward‐selection multivariable logistic regression.

**Results:**

Compared with survivors, patients in the AD group showed greater inflammatory activation, more pronounced coagulation disturbances, and higher plasma viral loads. While overall circulating Tfh frequencies were elevated in AD patients, subset profiling demonstrated a clear bias toward Tfh2 expansion together with contraction of Tfh1 and Tfh17 populations. Among these subsets, Tfh2 had the best discriminatory value for mortality (AUC = 0.724). Lower Tfh1 and Tfh17 frequencies were associated with poorer 28‐day survival. Viral RNA levels were positively related to total Tfh and Tfh2 frequencies and negatively related to Tfh17. After adjustment for age, HLH status, and viral load, Tfh1 remained an independent correlate of acute‐phase mortality.

**Conclusions:**

Acute SFTSV infection is associated with substantial remodeling of the circulating Tfh compartment. Expansion of Tfh2 alongside reduction of Tfh1 and Tfh17 was linked to heavier viral burden and worse clinical outcome. The independent association between lower Tfh1 frequency and mortality suggests that Tfh profiling may be useful for early risk assessment and offers mechanistic insight into defective humoral immunity in severe SFTS.

## 1. Introduction

Severe fever with thrombocytopenia syndrome (SFTS) is a recently discovered disease transmitted by ticks, caused by the SFTS virus (SFTSV), a member of the Bandavirus genus within the Phenuiviridae family [1, 2]. Since it was first described in China in 2009, cases have also been documented in Vietnam, South Korea, and Japan, and reported fatality rates generally range from 10% to 30% [1, 3, 4]. Typical manifestations include fever, thrombocytopenia, leukopenia, and multisystem involvement, whereas severe disease may progress to neurologic complications, disseminated coagulation disorders, and multiple‐organ failure [1, 5]. Although supportive management has improved, no antiviral treatment with proven efficacy is currently available, and the mechanisms driving severe disease remain only partly understood. Evidence is accumulating that highlights the importance of immune dysregulation in the development of SFTS [6, 7].

Protective immunity against SFTSV depends on coordinated innate and adaptive immune responses. In the early stage of infection, excessive production of IL‐6, TNF‐α, and IFN‐γ can trigger a cytokine storm and contribute to tissue damage [8]. Meanwhile, impaired antiviral antibody production and delayed viral clearance have been documented in fatal cases, suggesting a defective humoral immune response [9, 10]. However, the immunological mechanisms governing this humoral dysfunction remain poorly defined.

T follicular helper (Tfh) cells are a specialized CD4^+^ T‐cell subset defined by CXCR5 expression and their functional role in germinal centers, where they support B‐cell differentiation, somatic hypermutation, and affinity maturation [11]. Dysregulation of Tfh differentiation or function has been implicated in various viral infections, where excessive or skewed Tfh responses are associated with poor neutralizing antibody quality and uncontrolled viral replication [12–14]. Tfh cells can be categorized into three primary subsets based on chemokine receptor expression: Tfh1, Tfh2, and Tfh17, each varying in their capacity to enhance B‐cell differentiation and antibody production [15].

Recent studies have shown that SFTS patients exhibit profound lymphopenia, impaired B‐cell maturation, and reduced neutralizing antibody titers, thereby suggesting that Tfh dysfunction may contribute to defective antibody responses [7, 16]. However, research on the properties and roles of circulating Tfh cells in the context of SFTSV infection is lacking. Studying the response of Tfh subsets during acute infections might reveal important insights into the processes of immune dysregulation and the progression of diseases.

Therefore, in this research, we thoroughly examined the clinical features, lab profiles, and circulating Tfh subsets in SFTS patients. Additionally, we analyzed the relationship between Tfh subset distribution, viral load, and clinical outcomes to understand the potential impact of Tfh cell dysregulation on SFTS pathogenesis.

## 2. Methods

### 2.1. Patients

This study was conducted in accordance with our previously published protocols for patient enrollment, clinical classification, and laboratory assessment in SFTS, with minor modifications to address the objectives of the present cohort study [17]. Between May 2023 and October 2023, Tongji Hospital at Tongji Medical College, Huazhong University of Science and Technology in Wuhan, China, enrolled 79 patients with laboratory‐confirmed SFTS consecutively. The diagnosis of SFTS was made based on an acute feverish illness with a low platelet count and confirmed by detecting SFTSV RNA in plasma using the polymerase chain reaction. The acute phase was defined by a body temperature >38.5°C, a platelet count <150 × 10^9^/L, and the presence of nonspecific symptoms such as fatigue, myalgia, headache, or gastrointestinal manifestations. According to the clinical outcome during the acute phase, patients were classified into acute‐phase survivors (AS) and acute‐phase deceased (AD) groups.

### 2.2. Clinical Data Collection and Laboratory Measurements

Clinical information was obtained from Tongji Hospital’s electronic medical records using previously outlined standardized methods [17]. The collected data covered demographic traits, coexisting diseases, clinical presentations, lab findings, complications, treatment methods, and outcomes. Upon admission, all patients underwent a comprehensive laboratory evaluation. Peripheral blood samples for flow cytometry and SFTSV RNA quantification were collected on the day of hospital admission at the first available time point during the acute phase (baseline sampling) prior to outcome ascertainment. Corticosteroids, when administered, were initiated after the admission‐day baseline blood sampling used for flow cytometry. Hematological parameters were measured using an automated hematology analyzer (SYSMEX XE2100, Sysmex Corporation, Japan). Biochemical indices reflecting liver function, renal function, and inflammatory status were assessed using a fully automated biochemical analyzer (Roche Diagnostics). Coagulation parameters were determined with a STAGO automated coagulation analyzer. Serum cytokines were quantified using a solid‐phase two‐site chemiluminescent immunometric assay on an IMMULITE 1000 analyzer (Siemens). Clinical outcomes were monitored for 28 days from the symptom onset.

### 2.3. Circulating Tfh Cells Assessed by Flow Cytometry

The analysis of circulating Tfh cells and their subsets was conducted via flow cytometry, utilizing previously described staining and gating approaches [17]. Briefly, 100 μL of peripheral whole blood was incubated with fluorochrome‐conjugated monoclonal antibodies, including anti‐PD‐1‐PE, anti‐ICOS‐PE/Cy7, anti‐CCR6‐PerCP, anti‐CXCR5‐APC, anti‐CD4‐APC‐Cy7, anti‐CXCR3‐V450, anti‐CD45‐V500, anti‐CD8‐BV605, and anti‐CD45RA‐FITC (all from BD Pharmingen). Corresponding isotype controls were included to ensure staining specificity. After incubation for 20 min at room temperature in the dark, erythrocytes were lysed using a commercial lysing solution. After washing, the cells were resuspended in phosphate‐buffered saline and analyzed with a FACSCanto flow cytometer by BD Biosciences. PD‐1^+^ Tfh cells were quantified as the PD‐1–positive fraction within the cTfh gate. ICOS was included as an auxiliary activation marker for visualization but was not used to define PD‐1 positivity.

### 2.4. Quantification of SFTSV RNA by RT‐qPCR

According to the manufacturer’s instructions, plasma viral RNA was extracted with a commercial RNA isolation kit. Quantification of SFTSV RNA was performed using a TaqMan‐based real‐time quantitative reverse transcription PCR (RT‐qPCR) assay following previously reported protocols [17]. RT‐qPCR reactions were performed in triplicate for each sample.

### 2.5. Statistical Analysis

Before the analysis, data distribution was evaluated. Depending on what was appropriate, continuous variables were represented as mean ± standard deviation or median with interquartile range, and comparisons were made using the Student’s *t*‐test or the Mann–Whitney *U* test. The presentation of categorical variables involved counts and percentages, and comparisons were performed using the *χ* ^2^ test or Fisher’s exact test. For more than two groups, a one‐way ANOVA with Tukey’s post hoc adjustment was utilized. The prognostic performance of circulating Tfh subsets was assessed using receiver operating characteristic (ROC) curve analysis, which involved calculating the area under the curve. Survival differences were analyzed using Kaplan–Meier curves and the log‐rank test, with optimal cutoff values determined by ROC analysis. Associations between Tfh subsets and viral RNA load were assessed using Spearman’s rank correlation. To assess potential collinearity among Tfh‐related variables, correlation analysis was performed, and highly correlated variables were not included in the same multivariable model. Independent predictors of acute‐phase mortality were then identified using forward‐selection multivariable logistic regression. GraphPad Prism Version 8.0 and SPSS Version 22.0 were used for statistical analyses, with a two‐tailed *p*‐value of less than 0.05 being deemed statistically significant.

## 3. Results

### 3.1. Demographics and Clinical Characteristics of SFTS Patients

In this research, 79 patients diagnosed with SFTSV infection were enrolled, including 33 males and 46 females, with a median age of 64. Among them, 49 patients survived during the acute phase (AS group), whereas 30 patients died (AD group). The participants’ clinical characteristics are shown in Table 1. In SFTS patients, the two groups did not differ significantly in terms of sex distribution or symptoms. The AD group exhibited a higher occurrence of consciousness disorders, a more frequent use of continuous renal replacement therapy, and a greater requirement for respiratory support. Just 11.4% of patients mentioned having been bitten by a tick, while 17.7% reported engaging in field activities. In addition, the duration of hospital admission was significantly shorter in the AD group compared with the AS group. The proportion of patients complicated with hemophagocytic lymphohistiocytosis (HLH) was also higher in the AD group than in the AS group.

**Table 1:** Baseline demographic and clinical characteristics of SFTS patients on admission.

Parameters	Total (*n* = 79)	AS group (*n* = 49)	AD group (*n* = 30)	*p* value
Age, *y*, mean ± SD	64.684 ± 9.134	62.633 ± 9.169	68.033 ± 8.156	0.008
Sex				
Female, number (%)	46 (58.2)	28 (57.1)	18 (60.0)	0.988
Male, number (%)	33 (41.8)	21 (42.9)	12 (40.0)	—
Symptoms, number (%)				
Fever	73 (92.4)	47 (95.9)	26 (86.7)	0.194
Weakness	34 (43.0)	20 (40.8)	14 (46.7)	0.783
Muscular soreness	6 (7.6)	5 (10.2)	1 (3.3)	0.399
Inappetence	22 (27.8)	13 (26.5)	9 (30.0)	0.940
Nausea	23 (29.1)	13 (26.5)	10 (33.3)	0.696
Vomiting	26 (32.9)	16 (32.7)	10 (33.3)	>0.999
Abdominal pain	11 (13.9)	8 (16.3)	3 (10.0)	0.519
Diarrhea	39 (49.4)	24 (49.0)	15 (50.0)	>0.999
Headache and dizziness	23 (29.1)	13 (26.5)	10 (33.3)	0.696
Consciousness disorder	22 (27.8)	8 (16.3)	14 (46.7)	0.008
Comorbidities, number (%)				
Hypertension	24 (30.4)	15 (30.6)	9 (30.0)	>0.999
Coronary heart disease	10 (12.7)	5 (10.2)	5 (16.7)	0.492
Diabetes mellitus	7 (8.9)	4 (8.2)	3 (10.0)	>0.999
COPD	4 (5.1)	3 (6.1)	1 (3.3)	>0.999
Tuberculosis	2 (2.5)	2 (4.1)	0 (0.0)	0.523
Therapy, number (%)				
Corticosteroid	14 (17.7)	9 (18.4)	5 (16.7)	>0.999
CRRT	10 (12.7)	2 (4.1)	8 (26.7)	0.005
Respiratory support	5 (6.3)	0 (0.0)	5 (16.7)	0.006
History of tick bite, number (%)	9 (11.4)	7 (14.3)	2 (6.7)	0.470
History of field activities, number (%)	14 (17.7)	9 (18.4)	5 (16.7)	>0.999
Time from onset to admission, *d*, Median [Q1–Q3]	6.0 (5.0–7.0)	7.0 (5.0–8.0)	5.0 (4.0–7.0)	0.125
Duration of hospital admission, Median [Q1–Q3]	7.0 (3.0–11.0)	9.0 (7.0–13.0)	3.0 (2.0–4.0)	<0.001
Complicated with HLH, number (%)	34 (43.0)	16 (32.7)	18 (60.0)	0.032

*Note:* The data were compared between the survival and deceased groups.

Abbreviations: COPD, chronic obstructive pulmonary disease; CRRT, continuous renal replacement therapy; HLH, hemophagocytic lymphohistiocytosis; Q, quartile.

### 3.2. Differences in Laboratory Parameters Between Surviving and Deceased Patients With SFTS

The laboratory findings on admission are shown in Table 2. The AD group exhibited significantly reduced lymphocyte counts and platelet counts compared with those of the AS group. Liver injury markers were elevated in the AD group, including ALT, AST, LDH, and ALP, along with increased total and direct bilirubin. Renal function impairment was evident, with higher urea levels, decreased HCO_3_^−^, and lower eGFR in the AD group. Inflammatory markers were significantly higher in the AD group, with higher hsCRP, ferritin, and PCT. Coagulation abnormalities were prominent in the AD group, including prolonged PT, APTT, and TT, as well as increased D‐dimer levels. Furthermore, the AD group showed a significant reduction in fibrinogen levels compared to the AS group. Additionally, the cytokine profile indicated that the AD group exhibited notably increased levels of IL‐10, IL‐8, IL‐1β, IL‐2R, IL‐6, and TNF‐α relative to those of the AS group.

**Table 2:** Comparison of laboratory parameters between surviving and deceased SFTS patients on admission.

Parameter	Total (*n* = 79)	AS group (*n* = 49)	AD group (*n* = 30)	*p*‐Value
Blood routine indicators				
WBC, ×10^9^/L, median (Q1–Q3)	3.34 (1.75–6.42)	3.34 (1.74–6.44)	3.34 (1.78–6.04)	0.888
Neutrophiles, ×10^9^/L, median (Q1–Q3)	2.36 (1.03–4.18)	2.37 (0.98–4.25)	2.23 (1.26–4.09)	0.671
Lymphocytes, ×10^9^/L, median (Q1–Q3)	0.63 (0.33–1.03)	0.75 (0.37–1.24)	0.48 (0.22–0.92)	0.045
Monocytes, ×10^9^/L, median (Q1–Q3)	0.15 (0.08–0.33)	0.17 (0.09–0.46)	0.13 (0.05–0.24)	0.147
RBC, ×10^12^/L, median (Q1–Q3)	4.14 (3.67–4.61)	4.26 (3.64–4.61)	4.07 (3.67–4.61)	0.621
Hemoglobin, g/L	127.090 (22.725)	127.65 (21.49)	126.14 (25.03)	0.787
Platelet, ×10^9^/L, median (Q1–Q3)	44.00 (30.00–56.40)	50.00 (39.00–60.00)	37.00 (26.00–49.50)	0.029
Blood biochemistry indicators				
ALT, U/L, median (Q1–Q3)	74.50 (42.00–150.75)	55.50 (38.25–116.25)	109.50 (59.50–198.50)	0.027
AST, U/L, median (Q1–Q3)	221.00 (93.25–379.75)	153.00 (82.75–285.00)	337.50 (190.50–633.50)	0.001
LDH, U/L, median (Q1–Q3)	729.00 (463.50–1078.50)	655.00 (422.50–919.75)	901.50 (636.50–1425.25)	0.004
ALP, U/L, median (Q1–Q3)	71.00 (55.25–99.50)	65.00 (52.00–74.25)	80.50 (68.00–114.50)	0.005
Total protein, g/L, median (Q1–Q3)	60.75 (57.58–63.28)	61.10 (58.45–63.70)	60.50 (57.58–62.35)	0.361
Albumin, g/L, median (Q1–Q3)	33.05 (30.60–35.38)	33.15 (30.70–35.98)	32.95 (29.50–34.08)	0.143
Globulin, g/L median (Q1–Q3)	27.95 (25.95–29.75)	27.85 (25.58–29.85)	27.95 (26.90–29.30)	0.466
TBIL, μmol/L, median (Q1–Q3)	7.85 (5.83–11.83)	7.30 (5.60–11.90)	9.25 (6.30–10.18)	0.231
DBIL, μmol/L, median (Q1–Q3)	4.20 (2.88–6.15)	3.85 (2.70–5.83)	4.75 (3.43–6.15)	0.326
IBIL, μmol/L median (Q1–Q3)	3.15 (1.90–5.00)	3.20 (2.00–4.70)	2.60 (1.65–5.08)	0.618
Urea, mmol/L, median (Q1–Q3)	6.10 (4.73–8.18)	5.65 (4.30–7.40)	7.05 (6.03–9.50)	0.004
Uric acid, μmol/L, median (Q1–Q3)	249.50 (201.50–321.25)	249.50 (197.75–284.25)	253.00 (220.25–386.75)	0.194
Creatinine, μmol/L, median (Q1–Q3)	84.00 (68.00–108.50)	77.50 (66.00–104.50)	89.00 (68.75–116.00)	0.223
HCO_3_^−^, mmol/L, median (Q1–Q3)	20.35 (17.83–21.98)	20.70 (18.95–22.63)	18.60 (16.70–20.50)	0.004
eGFR, mL/min/1.73 m^2^, mean (SD)	68.45 (22.95)	73.87 (21.63)	59.77 (22.67)	0.009
Amylopsin, U/L, median (Q1–Q3)	82.00 (49.00–127.00)	82.50 (48.25–115.50)	80.00 (54.50–134.00)	0.361
Lipase, IU/L, median (Q1–Q3)	192.50 (95.40–329.50)	161.30 (82.70–248.58)	265.20 (115.10–341.95)	0.120
Triglyceride, mmol/L, median (Q1–Q3)	2.22 (1.45–3.05)	2.33 (1.73–3.15)	1.80 (1.31–2.62)	0.109
Total cholesterol, mmol/L, median (Q1–Q3)	3.03 (2.48–3.62)	3.16 (2.49–3.58)	2.77 (2.26–3.62)	0.353
Coagulation markers				
TT, s, median (Q1–Q3)	26.20 (21.95 −37.80)	24.10 (21.40–27.75)	32.55 (25.18–51.48)	0.003
PT, s, median (Q1–Q3)	12.80 (12.15–13.50)	12.50 (11.80–13.00)	13.50 (12.45–14.60)	0.002
APTT, s, median (Q1–Q3)	57.90 (44.70–64.78)	49.55 (42.03–61.53)	63.85 (55.03–82.88)	<0.001
Fibrinogen, g/L, median (Q1–Q3)	2.58 (2.32–3.02)	2.78 (2.40–3.10)	2.39 (1.90–2.72)	0.006
D‐dimer, μg/mL FEU, median (Q1–Q3)	2.62 (1.60–6.08)	2.08 (1.16–3.39)	6.46 (3.60–10.14)	<0.001
Inflammatory indicators				
hsCRP mg/L, median (Q1–Q3)	3.70 (1.40 −11.60)	2.60 (1.23–6.80)	5.60 (2.90–18.40)	0.014
Ferritin, μg/L, median (Q1–Q3)	8740.85 (3091.03–19508.00)	5752.60 (2046.10–12089.00)	22989.00 (9613.55–47919.50)	<0.001
PCT, ng/mL, median (Q1–Q3)	0.26 (0.12–0.66)	0.21 (0.08–0.36)	0.44 (0.17–1.28)	0.004
Cytokine indicators				
IL‐1β, pg/mL, median (Q1–Q3)	5.00 (5.00–9.90)	5.00 (5.00–8.40)	6.45 (5.00–32.75)	0.027
IL‐2R, U/mL, median (Q1–Q3)	1237.50 (911.25–1773.75)	1123.00 (911.25–1341.50)	1726.50 (975.25–2494.25)	0.027
IL‐8, pg/mL, median (Q1–Q3)	24.20 (14.10–47.75)	20.90 (14.05–33.45)	52.10 (16.60–409.25)	0.025
IL‐10, pg/mL, median (Q1–Q3)	60.00 (21.10–138.00)	28.70 (15.25–89.50)	179.50 (66.53–246.25)	<0.001
TNF‐α, pg/mL, median (Q1–Q3)	22.10 (16.45–38.95)	19.10 (14.90–27.10)	38.95 (21.50–126.50)	<0.001
IL‐6, pg/mL, median (Q1–Q3)	35.23 (14.21–70.92)	28.11 (11.55–39.34)	100.10 (39.32–205.90)	<0.001

*Note:* The data were compared between the survival and deceased groups.

Abbreviations: ALP, alkaline phosphatase; ALT, alanine aminotransferase; APTT, activated partial thromboplastin time; AST, aspartate aminotransferase; DBIL, direct bilirubin; eGFR, estimated glomerular filtration rate; FEU, fibrinogen equivalent unit; hsCRP, high‐sensitivity C‐reactive protein; IBIL, indirect bilirubin; IL, interleukin; LDH, lactate dehydrogenase; PCT, procalcitonin; PT, prothrombin time; RBC, red blood cell; TBIL, total bilirubin; TNF‐α, tumor necrosis factor‐α; TT, thrombin time; WBC, white blood cell.

### 3.3. Alterations of Circulating Tfh Cells in SFTS Patients

**Figure 1:**
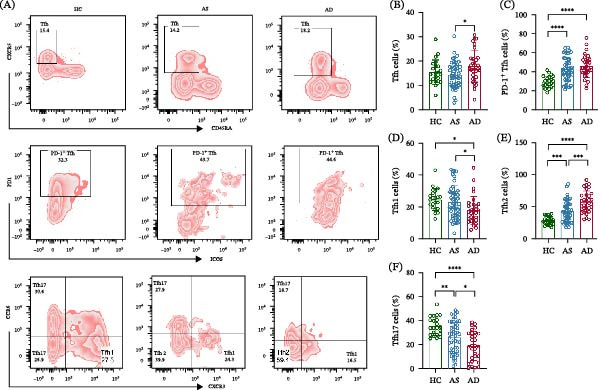
Circulating Tfh cells and subsets in patients with SFTSV infection. (A) Representative flow cytometry plots showing Tfh cells, PD‐1^+^ Tfh cells, Tfh1 cells, Tfh2 cells, and Tfh17 cells. (B) The frequency of total Tfh cells among CD4^+^ T cells; (C) The percentage of PD‐1^+^ Tfh cells among Tfh cells. PD‐1 positivity was evaluated within the CXCR5^+^ circulating Tfh (cTfh) gate (see Supporting Information 1 Figure S1 for the full gating strategy). (D–F) The frequency of Tfh1, Tfh2, and Tfh17 subsets among Tfh cells. Comparisons were performed among healthy controls (HC, *n* = 22), acute survivors (AS, *n* = 49), and acute deceased (AD, *n* = 30). Data are shown as mean ± SD. Statistical significance was determined using one‐way ANOVA with Tukey’s multiple‐comparisons test. Each participant contributed one sample analyzed once (one experiment/measurement per participant; no technical replicates). AS, patients who survived at the acute phase of disease; AD, patients who subsequently deceased at the acute phase of disease.  ^∗^ *p* < 0.05,  ^∗∗^ *p* < 0.01,  ^∗∗∗^ *p* < 0.001,  ^∗∗∗∗^ *p* < 0.0001. Descriptive statistics (mean ± SD) for panels B–F are provided in Supporting Information 2 Table S1.

In SFTS patients, significant alterations were observed in Tfh cells and their subsets through flow cytometry (Figure 1A). There was no difference in the overall frequency of total Tfh cells between SFTS patients (AS and AD) and healthy controls (HC); however, it was significantly elevated in the deceased group (AD) relative to the surviving group (AS) (Figure 1B). Similarly, PD‐1^+^ Tfh cells were markedly increased in both AS and AD patients compared with HCs, with no difference between the two patient groups (Figure 1C). We next analyzed the distribution of Tfh subsets. In the AD group, Tfh1 cells were notably lower than those in both the HC and AS groups (Figure 1D). Conversely, Tfh2 cells were higher in SFTS patients than in HCs and increased even more in the AD group compared to the AS group (Figure 1E). Moreover, Tfh17 cells were reduced in SFTS patients (AS and AD) compared with HC and were further decreased in the AD group relative to the AS group (Figure 1F). Descriptive statistics (mean ± SD) for Figure 1B–F are provided in Supporting Information 2 Table S1.

Given the imbalance in HLH prevalence between AS and AD groups, we performed a stratified analysis restricted to non‐HLH patients. Notably, the key Tfh subset alterations persisted in this subgroup, characterized by increased Tfh2 and decreased Tfh1 and Tfh17 frequencies in AD patients compared with AS patients (Supporting Information 2 Table S2), indicating that these changes were not merely driven by HLH status. To further assess whether variability in the admission‐day baseline sampling timing influenced circulating Tfh measurements, we performed a sensitivity analysis comparing patients admitted early (≤5 days from symptom onset) with those admitted later (>5 days). No significant differences were observed in total Tfh, PD‐1^+^ Tfh, Tfh1, Tfh2, or Tfh17 between the two groups (Supporting Information 2 Table S3), suggesting that sampling timing is unlikely to have materially affected the distribution of circulating Tfh subsets.

### 3.4. Predictive Value of Circulating Tfh Subsets for Disease Outcome in SFTS

**Figure 2:**
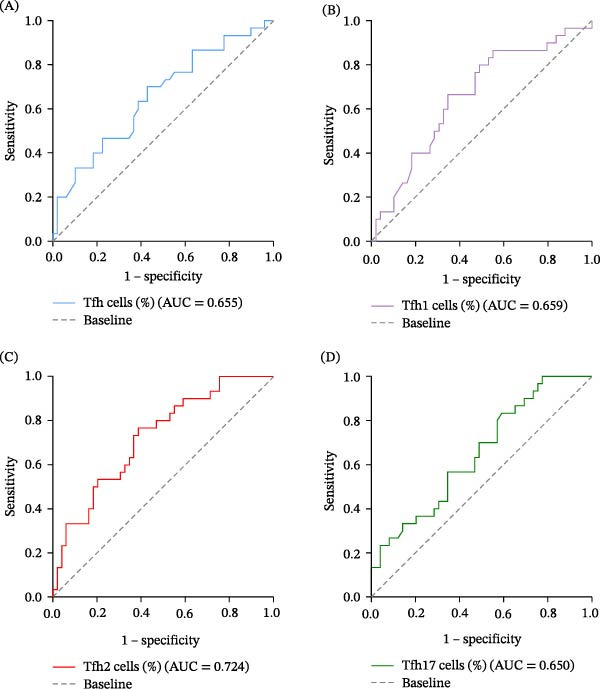
ROC analysis of circulating Tfh subsets in SFTS patients. ROC curves were used to assess the predictive value of (A) Tfh cells, (B) Tfh1 cells, (C) Tfh2 cells, and (D) Tfh17 cells for distinguishing between AS (*n* = 49) and AD (*n* = 30) groups. AUC values with 95% confidence intervals (CI) were calculated, and optimal cutoff values were determined by ROC analysis. Each participant contributed one sample analyzed once. AUC, area under the curve; HC, healthy control; ROC, receiver operating characteristic; SFTS, severe fever with thrombocytopenia syndrome; Tfh, T follicular helper.

To evaluate the prognostic significance of circulating Tfh cells in SFTS, ROC curve analysis was performed to discriminate the AS group from the AD group (Table 3). The overall Tfh population had a moderate predictive capability for poor outcomes, with an AUC of 0.655 (95% CI: 0.528–0.773), a sensitivity of 0.700, and a specificity of 0.571 at a threshold of 16.00 (Figure 2A). Within the Tfh subsets, the Tfh1 subset produced an AUC of 0.659 (95% CI: 0.523–0.761), with a sensitivity of 0.653 and a specificity of 0.667 (Figure 2B). Tfh2 cells displayed the highest discriminative performance (AUC = 0.724, 95% CI: 0.590–0.812), with sensitivity = 0.767 and specificity = 0.612 at a cutoff of 45.90, while Tfh17 cells exhibited relatively lower accuracy (AUC = 0.650, 95% CI: 0.515–0.766) but the highest specificity (0.833) at a cutoff of 30.60 (Figure 2C,D). Among the analyzed parameters, the Tfh2 frequency exhibited the highest AUC value (0.724), whereas the Tfh1 and Tfh17 subsets showed moderate predictive performance.

**Table 3:** The predictive ability of risk markers for poor prognosis in SFTS patients.

Parameters	AUC (95% CI)	Sensitivity	Specificity	Cutoff value
AS vs. AD				
Tfh	0.655 (0.528–0.773)	0.700	0.571	16.000
Tfh1	0.659 (0.523–0.761)	0.653	0.667	19.400
Tfh2	0.724 (0.590–0.812)	0.767	0.612	45.900
Tfh17	0.650 (0.515–0.766)	0.408	0.833	30.600

Abbreviation: AUC, area under the curve.

To determine whether circulating Tfh subsets independently predict acute‐phase mortality in SFTS, we performed forward‐selection multivariable logistic regression including age, HLH status, viral RNA load, total Tfh, PD‐1^+^ Tfh, Tfh1, and Tfh2 frequencies. As shown in Table 4, Tfh1 frequency remained independently associated with acute‐phase mortality (OR = 0.90, 95% CI: 0.82–0.98, *p* = 0.012), together with age and viral RNA load.

**Table 4:** Multivariable logistic regression for acute‐phase mortality in SFTS patients.

Parameters	*p* (univariable)	95% CI (univariable)	*p* (multivariable)	95% CI (multivariable)
Age (years)	0.013	1.07 (1.02–1.14)	0.004	1.15 (1.05–1.27)
HLH status (yes vs. no)	0.019	3.09 (1.20–7.95)	—	—
Log_10_ (SFTSV RNA)	<0.001	4.85 (2.43–9.68)	<0.001	7.99 (2.91–21.99)
Total Tfh (% of CD4^+^ T cells)	0.012	2.95 (1.26–6.89)	—	—
PD‐1^+^ Tfh (% of Tfh)	0.256	1.25 (0.85–1.83)	—	—
Tfh1 (% of Tfh)	0.028	0.54 (0.32–0.94)	0.012	0.90 (0.82–0.98)
Tfh2 (% of Tfh)	0.001	1.66 (1.22–2.27)	—	—

*Note:* Univariable and forward‐selection multivariable logistic regression for the outcome acute‐phase deceased (AD = 1) vs. acute‐phase survivor (AS = 0). Two‐sided *p* < 0.05 is considered statistically significant.

### 3.5. Prognostic Value of Tfh Subsets

**Figure 3:**
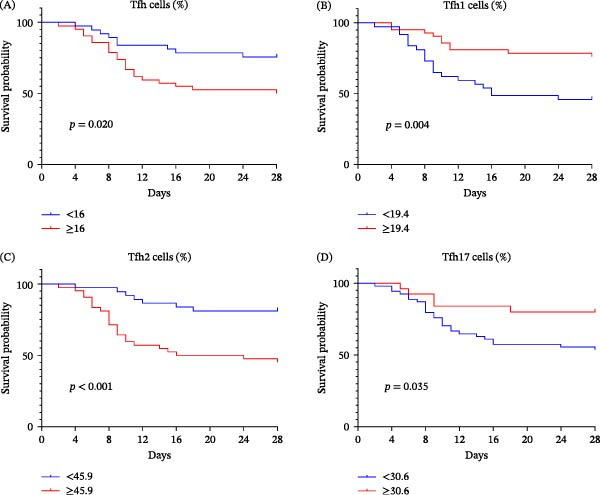
Kaplan–Meier survival curves showing the 28‐day survival rates with circulating Tfh subsets. (A) Kaplan–Meier curves of 28‐day survival stratified by the frequency of total Tfh cells among CD4^+^ T cells. (B–D) Kaplan–Meier curves stratified by the frequency of Tfh1, Tfh2, and Tfh17 among Tfh cells, respectively. Patients were dichotomized using ROC‐derived optimal cutoff values, and survival differences were evaluated using the log‐rank test. All analyses were based on admission‐day baseline samples (total *n* = 79), with one measurement per participant. SFTS, severe fever with thrombocytopenia syndrome; Tfh, T follicular helper.

The Kaplan–Meier survival analysis indicated that increased levels of total Tfh cells (*p* = 0.020) and Tfh2 cells (*p*  < 0.001) were notably linked to a worse prognosis in SFTS patients (Figure 3A,C). In contrast, reduced proportions of Tfh1 cells and Tfh17 cells were also linked to unfavorable outcomes (Figure 3B,D).

### 3.6. Correlation of Tfh Subsets With SFTSV RNA Viral Load in Patients With SFTS

**Figure 4:**
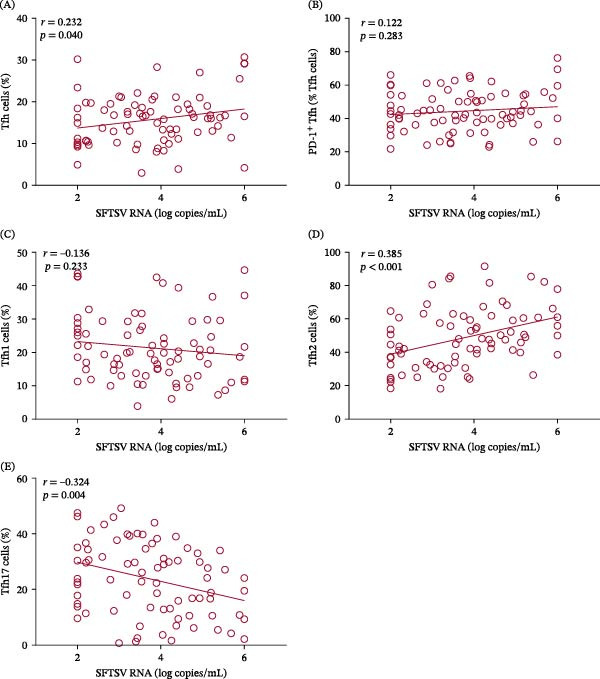
Correlation of Tfh subsets with SFTSV RNA load. Correlation between SFTSV RNA load and (A) the frequency of total Tfh cells among CD4^+^ T cells, (B) the frequency of PD‐1^+^ Tfh among Tfh cells, (C) the frequency of Tfh1 among Tfh cells, (D) the frequency of Tfh2 among Tfh cells, or (E) the frequency of Tfh17 among Tfh cells. Associations were assessed using Spearman’s rank correlation (two‐tailed). Correlations were calculated using admission‐day baseline samples (*n* = 79). SFTSV RNA quantification by RT‐qPCR was performed in triplicate per sample, and one admission‐day baseline sample was analyzed per participant. SFTS, severe fever with thrombocytopenia syndrome; Tfh, T follicular helper.

Given the altered distribution of Tfh subsets in SFTS patients, we next examined their association with the SFTSV RNA viral load. The correlation analysis showed a statistically significant positive link between viral load and the frequency of both total Tfh cells and Tfh2 cells (Figure 4A,D). Conversely, there was a negative correlation between Tfh17 cells and the viral load (Figure 4E). A significant correlation between viral load and PD‐1^+^ Tfh cells (Figure 4B) or Tfh1 cells (Figure 4C) was not observed.

## 4. Discussion

In this study, we provide the detailed characterization of circulating Tfh subsets in SFTS and demonstrate a marked imbalance in Tfh differentiation associated with disease severity. Although total circulating Tfh cells were increased in deceased patients, the most prominent abnormality was a shift toward a Tfh2‐like profile, accompanied by significant reductions in Tfh1 and Tfh17 subsets. Tfh functional heterogeneity is well established, with Tfh2 cells preferentially promoting extrafollicular plasmablast differentiation and low‐affinity antibody production, whereas Tfh1 and Tfh17 subsets more effectively support germinal‐center maturation and high‐affinity class‐switched antibody responses [11, 15, 18, 19]. The predominance of Tfh2‐like cells together with the contraction of Tfh1 and Tfh17 subsets in fatal SFTS therefore provides a mechanistic explanation for the delayed seroconversion, attenuated neutralizing antibody responses, and impaired B‐cell maturation repeatedly observed in severe disease [9, 10, 16].

Beyond subset redistribution, SFTS patients exhibited substantial upregulation of PD‐1 on circulating Tfh cells, indicating marked Tfh activation during acute SFTSV infection. Notably, PD‐1^+^ cTfh frequencies were increased in both survivors and deceased patients compared with those in HC, suggesting that PD‐1 upregulation reflects a shared infection‐driven activation signature rather than a mortality‐specific marker. While expansion of activated PD‐1^+^ cTfh cells has been linked to effective antibody responses in certain contexts, such as influenza vaccination in healthy individuals [20], in severe infection with a high antigen burden and systemic hyperinflammation, sustained PD‐1 expression may instead be associated with qualitative alterations of Tfh help and compromised germinal‐center support. PD‐1–associated Tfh dysfunction has been described in acute and chronic viral infections and has been reported to impair IL‐21 production, germinal‐center support, and affinity maturation [12, 13, 21]. The inflammatory milieu characteristic of acute SFTSV infection, marked by elevated IL‐6, IL‐10, TNFα, and dysregulated cytokine signatures, may further alter Tfh differentiation and suppress germinal‐center‐directed helper activity [6, 7, 22]. IL‐6 and IL‐10 in particular are critical regulators of Tfh lineage commitment and can perturb germinal‐center architecture or skew Tfh functionality in settings of heightened inflammation [14, 23, 24].

Importantly, Tfh subset distribution in our cohort correlated with viral RNA load, suggesting a direct link between Tfh dysregulation and the failure to control viral replication. This idea aligns with findings in other viral infections, where a lack or delay in the development of functional virus‐specific circulating Tfh cells is linked to a higher viral load and more severe clinical symptoms [23, 25]. Mechanistically, a Tfh2‐skewed response is more likely to promote rapid extrafollicular plasmablast differentiation and early antibody output; however, such responses are often biased toward quantity rather than quality and may yield antibodies with lower affinity and suboptimal neutralizing capacity. In contrast, Tfh1 and Tfh17 subsets more effectively sustain germinal‐center reactions, enabling somatic hypermutation, affinity maturation, and class switching that generate high‐affinity neutralizing antibodies and durable memory—features typically required for efficient viral clearance [19, 26, 27]. Therefore, the increase in Tfh2‐like cells together with the contraction of Tfh1 and Tfh17 subsets provides a plausible mechanistic basis for inadequate neutralizing antibody development and subsequent uncontrolled viral proliferation. These findings align with established paradigms in viral immunology, in which disruption of the Tfh and B‐cell axis, including skewing toward dysfunctional Th2‐biased Tfh cells, is associated with poor humoral immunity and severe clinical outcomes in chronic or severe viral infections [23, 28, 29].

Given that HLH is known to profoundly affect T‐cell homeostasis [30], we carefully evaluated its potential confounding effect. Stratified analysis restricted to non‐HLH patients demonstrated that the imbalance of Tfh subsets persisted, indicating that these alterations are not merely epiphenomena of HLH but may represent intrinsic immune dysregulation associated with SFTS severity. Furthermore, multivariable analysis confirmed that Tfh1 remained independently associated with mortality after adjustment for the HLH status and other clinical variables. Nevertheless, residual confounding by HLH cannot be completely excluded.

Our study has several limitations. Circulating Tfh subsets may not fully represent lymphoid‐resident GC‐Tfh cells, and functional Tfh–B‐cell coculture assays as well as anti‐SFTSV binding/neutralizing antibody titers and antibody‐affinity measurements were not performed. Additionally, this was a single‐center cohort and requires multicenter validation. ROC‐derived cutoffs used for Kaplan–Meier stratification were derived from the same cohort and may be subject to overfitting; therefore, the optimal thresholds and their prognostic performance require validation in independent cohorts. Future studies integrating longitudinal antibody kinetics, single‐cell multi‐omics, and spatial immune profiling will be essential to delineate how Tfh subset dynamics shape antiviral and immunopathological responses in SFTSV infection.

Taken together, our results indicate that fatal SFTS is characterized by a qualitative imbalance of Tfh subsets, characterized by Tfh2‐like predominance, contraction of Tfh1 and Tfh17 populations, and elevated PD‐1 expression. This imbalance compromises germinal‐center dependent B‐cell maturation and antiviral antibody production. Circulating Tfh profiling therefore represents a promising immunologic marker to complement viral load‐based risk assessment and may inform future therapeutic strategies aimed at restoring balanced Tfh function or modulating the inflammatory environment to improve antiviral humoral immunity in SFTS.

## Author Contributions

**Danning Xu:** data curation, writing – original draft. **Wei Wei:** methodology. **Yun Wang:** visualization. **Ting Wang:** methodology, validation, data curation. **Rujia Chen:** investigation, resources. **Renren Ouyang:** resources. **Shiji Wu and Hongyan Hou:** writing – review and editing. **Feng Wang:** writing – review and editing, supervision.

## Funding

This study was funded by the project of the National Natural Science Foundation of China (Grant 82502807).

## Ethics Statement

This study was approved by the ethical committee of Tongji Hospital, Tongji Medical College, Huazhong University of Science and Technology (TJ‐IRB20230632). Before participating in the study, all individuals signed a written informed consent, and the study protocol complies with the Declaration of Helsinki’s ethical standards.

## Consent

Informed consent was secured from each participant involved in the study.

## Conflicts of Interest

The authors declare no conflicts of interest.

## Supporting Information

Additional supporting information can be found online in the Supporting Information section.

## Supporting information


**Supporting Information 1** Figure S1: Gating strategy for circulating Tfh (cTfh) cells.

**Supporting Information 2** Table S1: Descriptive statistics for circulating Tfh parameters in Figure 1B–F. Table S2: Sensitivity analysis restricted to non‐HLH patients. Table S3: Sensitivity analysis of circulating Tfh subsets according to timing of admission‐day baseline sampling.

## Data Availability

The data that support the findings of this study are available upon request from the corresponding author. The data are not publicly available due to privacy or ethical restrictions.

## References

[1] Yu X. J. , Liang M. F. , and Zhang S. Y. , et al.Fever With Thrombocytopenia Associated With a Novel Bunyavirus in China, New England Journal of Medicine. (2011) 364, no. 16, 1523–1532, 10.1056/NEJMoa1010095. 21410387 PMC3113718

[2] Liu Q. , He B. , Huang S.-Y. , Wei F. , and Zhu X.-Q. , Severe Fever With Thrombocytopenia Syndrome, an Emerging Tick-Borne Zoonosis, The Lancet Infectious Diseases. (2014) 14, no. 8, 763–772, 10.1016/S1473-3099(14)70718-2. 24837566

[3] Takahashi T. , Maeda K. , and Suzuki T. , et al.The First Identification and Retrospective Study of Severe Fever With Thrombocytopenia Syndrome in Japan, The Journal of Infectious Diseases. (2014) 209, no. 6, 816–827, 10.1093/infdis/jit603. 24231186 PMC7107388

[4] Kim K. H. , Yi J. , and Kim G. , et al.Severe Fever With Thrombocytopenia Syndrome, South Korea, 2012, Emerging Infectious Diseases. (2013) 19, no. 11, 1892–1894, 10.3201/eid1911.130792. 24206586 PMC3837670

[5] Casel M. A. , Park S. J. , and Choi Y. K. , Severe Fever With Thrombocytopenia Syndrome Virus: Emerging Novel Phlebovirus and Their Control Strategy, Experimental and Molecular Medicine. (2021) 53, no. 5, 713–722, 10.1038/s12276-021-00610-1. 33953322 PMC8178303

[6] Niu Y. , Liu Y. , and Huang L. , et al.Antiviral Immunity of Severe Fever With Thrombocytopenia Syndrome: Current Understanding and Implications for Clinical Treatment, Frontiers in Immunology. (2024) 15, 10.3389/fimmu.2024.1348836, 1348836. 38646523 PMC11026560

[7] Chen Y. , Miller H. , and Benlagha K. , et al.A Focus on the Mechanisms of Alteration in Host Lymphocyte Level Following Severe Fever With Thrombocytopenia Syndrome Virus (SFTSV) Infection, Journal of Inflammation Research. (2025) 18, 13265–13277, 10.2147/JIR.S531068. 41031180 PMC12477067

[8] Kwon J. S. , Kim J. Y. , and Jang C. Y. , et al.Effect of Severe Fever With Thrombocytopenia Syndrome Virus Genotype on Disease Severity, Viral Load, and Cytokines in South Korea, Open Forum Infectious Diseases. (2024) 11, no. 9, 10.1093/ofid/ofae508, ofae508. 39310272 PMC11414404

[9] Li J. C. , Ding H. , and Wang G. , et al.Dynamics of Neutralizing Antibodies Against Severe Fever With Thrombocytopenia Syndrome Virus, International Journal of Infectious Diseases. (2023) 134, 95–98, 10.1016/j.ijid.2023.05.018. 37247691

[10] Song P. , Zheng N. , and Liu Y. , et al.Deficient Humoral Responses and Disrupted B-Cell Immunity Are Associated With Fatal SFTSV Infection, Nature Communications. (2018) 9, no. 1, 10.1038/s41467-018-05746-9, 3328. PMC6102208 30127439

[11] Crotty S. , T Follicular Helper Cell Differentiation, Function, and Roles in Disease, Immunity. (2014) 41, no. 4, 529–542, 10.1016/j.immuni.2014.10.004. 25367570 PMC4223692

[12] Cubas R. A. , Mudd J. C. , and Savoye A. L. , et al.Inadequate T Follicular Cell Help Impairs B Cell Immunity During HIV Infection, Nature Medicine. (2013) 19, no. 4, 494–499, 10.1038/nm.3109. PMC3843317 23475201

[13] Raziorrouh B. , Sacher K. , and Tawar R. G. , et al.Virus-Specific CD4+ T Cells Have Functional and Phenotypic Characteristics of Follicular T-Helper Cells in Patients With Acute and Chronic HCV Infections, Gastroenterology. (2016) 150, no. 3, 696–706.e3, 10.1053/j.gastro.2015.11.005. 26584604

[14] Kaneko N. , Kuo H. H. , and Boucau J. , et al.Loss of Bcl-6-Expressing T Follicular Helper Cells and Germinal Centers in COVID-19, Cell. (2020) 183, no. 1, 143–157.e13, 10.1016/j.cell.2020.08.025. 32877699 PMC7437499

[15] Morita R. , Schmitt N. , and Bentebibel S. E. , et al.Human Blood CXCR5+CD4+ T Cells Are Counterparts of T Follicular Cells and Contain Specific Subsets that Differentially Support Antibody Secretion, Immunity. (2011) 34, no. 1, 108–121, 10.1016/j.immuni.2010.12.012. 21215658 PMC3046815

[16] Suzuki T. , Sato Y. , and Sano K. , et al.Severe Fever With Thrombocytopenia Syndrome Virus Targets B Cells in Lethal Human Infections, Journal of Clinical Investigation. (2020) 130, no. 2, 799–812, 10.1172/JCI129171. 31904586 PMC6994144

[17] Hou H. , Zou S. , and Wei W. , et al.Kinetics and Prognostic Significance of Laboratory Markers in Patients With Severe Fever With Thrombocytopenia Syndrome: Insight From a Comprehensive Analysis, The Journal of Infectious Diseases. (2024) 229, no. 6, 1845–1855, 10.1093/infdis/jiad426. 37804100

[18] Schmitt N. , Bentebibel S.-E. , and Ueno H. , Phenotype and Functions of Memory Tfh Cells in Human Blood, Trends in Immunology. (2014) 35, no. 9, 436–442, 10.1016/j.it.2014.06.002. 24998903 PMC4152409

[19] Crotty S. , T Follicular Helper Cell Biology: A Decade of Discovery and Diseases, Immunity. (2019) 50, no. 5, 1132–1148, 10.1016/j.immuni.2019.04.011. 31117010 PMC6532429

[20] Bentebibel S. E. , Khurana S. , and Schmitt N. , et al.ICOS(+)PD-1(+)CXCR3(+) T Follicular Helper Cells Contribute to the Generation of High-Avidity Antibodies following Influenza Vaccination, Scientific Reports. (2016) 6, no. 1, 10.1038/srep26494, 26494. 27231124 PMC4882544

[21] Jubel J. M. , Barbati Z. R. , Burger C. , Wirtz D. C. , and Schildberg F. A. , The Role of PD-1 in Acute and Chronic Infection, Frontiers in Immunology. (2020) 11, 10.3389/fimmu.2020.00487, 487. 32265932 PMC7105608

[22] Liu Z. , Xue X. , and Geng S. , et al.The Differences in Cytokine Signatures Between Severe Fever With Thrombocytopenia Syndrome (SFTS) and Hemorrhagic Fever With Renal Syndrome (HFRS), Journal of Virology. (2024) 98, no. 7, 10.1128/jvi.00786-24, e0078624. 38916398 PMC11265425

[23] Yu M. , Charles A. , and Cagigi A. , et al.Delayed Generation of Functional Virus-Specific Circulating T Follicular Helper Cells Correlates With Severe COVID-19, Nature Communications. (2023) 14, no. 1, 10.1038/s41467-023-37835-9, 2164. PMC10105364 37061513

[24] Ryg-Cornejo V. , Ioannidis L. J. , and Ly A. , et al.Severe Malaria Infections Impair Germinal Center Responses by Inhibiting T Follicular Helper Cell Differentiation, Cell Reports. (2016) 14, no. 1, 68–81, 10.1016/j.celrep.2015.12.006. 26725120

[25] Lu X. , Zhang X. , and Cheung A. K. L. , et al.Abnormal Shift in B Memory Cell Profile Is Associated With the Expansion of Circulating T Follicular Helper Cells via ICOS Signaling During Acute HIV-1 Infection, Frontiers in Immunology. (2022) 13, 10.3389/fimmu.2022.837921, 837921. 35222430 PMC8867039

[26] Song W. and Craft J. , T Follicular Helper Cell Heterogeneity, Annual Review of Immunology. (2024) 42, no. 1, 127–152, 10.1146/annurev-immunol-090222-102834. 38060987

[27] Foster W. S. , Lee J. L. , and Thakur N. , et al.Tfh Cells and the Germinal Center Are Required for Memory B Cell Formation and Humoral Immunity after ChAdOx1 nCoV-19 Vaccination, Cell Reports Medicine. (2022) 3, no. 12, 10.1016/j.xcrm.2022.100845, 100845. 36455555 PMC9663747

[28] Yin S. , Wang J. , and Chen L. , et al.Circulating Th2-Biased T Follicular Helper Cells Impede Antiviral Humoral Responses During Chronic Hepatitis B Infection Through Upregulating CTLA4, Antiviral Research. (2023) 216, 10.1016/j.antiviral.2023.105665, 105665. 37421985

[29] Chakhtoura M. , Fang M. , and Cubas R. , et al.Germinal Center T Follicular Helper (GC-Tfh) Cell Impairment in Chronic HIV Infection Involves c-Maf Signaling, PLoS Pathogens. (2021) 17, no. 7, 10.1371/journal.ppat.1009732, e1009732. 34280251 PMC8289045

[30] Jordan M. B. , Hemophagocytic Lymphohistiocytosis: A Disorder of T Cell Activation, Immune Regulation, and Distinctive Immunopathology, Immunological Reviews. (2024) 322, no. 1, 339–350, 10.1111/imr.13298. 38100247

