# Supplementary material for: T Follicular Helper Cell Immune Signatures Associated With Disease Severity in Severe Fever With Thrombocytopenia Syndrome
Source: J Immunol Res. 2026 Jun 23;2026:8984077. doi: 10.1155/jimr/8984077 (PMC13291557; doi:10.1155/jimr/8984077)
Supplement: Supplementary file 1 — Supporting Information 1 Figure S1: Gating strategy for circulating Tfh (cTfh) cells. [file JIMR-2026-8984077-s002.doc]

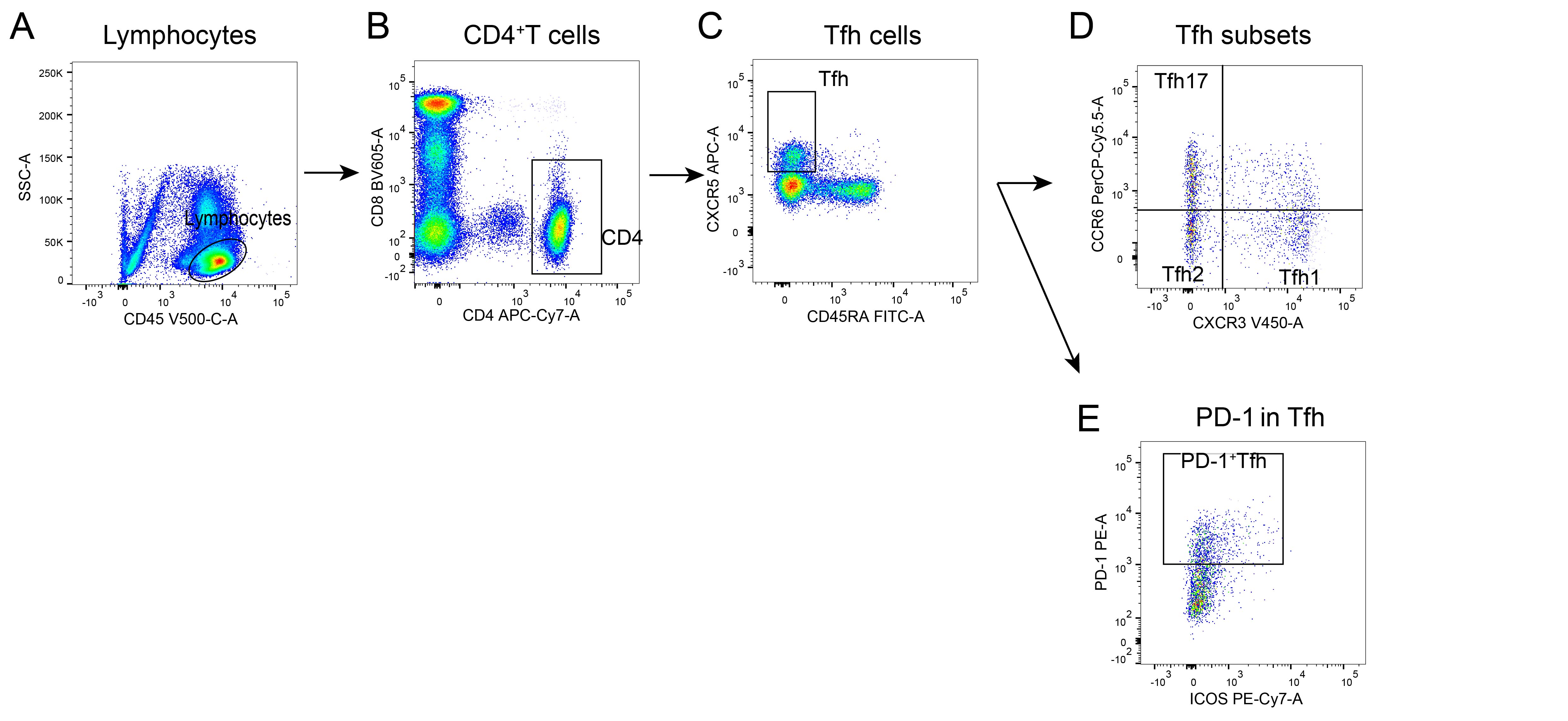
**Supplementary Figure S1. Gating strategy for circulating Tfh (cTfh) cells.** Representative flow cytometry plots show sequential gating of (A) lymphocytes, (B) CD4⁺ T cells, and (C) CXCR5⁺ circulating Tfh (cTfh) cells. From the cTfh gate, (D) Tfh subsets were identified based on CXCR3 and CCR6 expression (Tfh1, CXCR3⁺CCR6⁻; Tfh2, CXCR3⁻CCR6⁻; Tfh17, CXCR3⁻CCR6⁺), and (E) PD-1⁺ Tfh cells were quantified within the cTfh population. PD-1 positivity was evaluated within the CXCR5⁺ cTfh gate; ICOS is displayed for visualization and was not used to define PD-1⁺ gating. Representative plots are shown; each participant contributed one sample analyzed once.
