# Supplementary material for: T Follicular Helper Cell Immune Signatures Associated With Disease Severity in Severe Fever With Thrombocytopenia Syndrome
Source: J Immunol Res. 2026 Jun 23;2026:8984077. doi: 10.1155/jimr/8984077 (PMC13291557; doi:10.1155/jimr/8984077)
Supplement: Supplementary file 2 — Supporting Information 2 Table S1: Descriptive statistics for circulating Tfh parameters in Figure 1B–F. Table S2: Sensitivity analysis restricted to non‐HLH patients. Table S3: Sensitivity analysis of circulating Tfh subsets according to timing of admission‐day baseline sampling. [file JIMR-2026-8984077-s001.docx]

**Supplementary Table S1. Descriptive statistics for circulating Tfh parameters in Figure 1B–F**

| Parameter | HC (n=22) | AS (n=49) | AD (n=30) |
| --- | --- | --- | --- |
| Total Tfh (% of CD4⁺ T cells) | 15.52 ± 4.98 | 14.34 ± 5.32 | 18.00 ± 6.58 |
| PD-1⁺ Tfh (% of Tfh) | 30.15 ± 6.09 | 43.23 ± 12.23 | 46.43 ± 12.00 |
| Tfh1 (% of Tfh) | 24.91 ± 6.75 | 23.40 ± 10.02 | 18.24 ± 8.73 |
| Tfh2 (% of Tfh) | 28.45 ± 6.33 | 43.38 ± 16.05 | 57.63 ± 17.11 |
| Tfh17 (% of Tfh) | 35.73 ± 7.84 | 26.31 ± 12.97 | 19.32 ± 12.10 |
| Data are presented as mean ± SD. | | | |

**Supplementary Table S2. Sensitivity analysis restricted to non-HLH patients**

| Parameter | AS (non-HLH, n=33) | AD (non-HLH, n=12) | p value |
| --- | --- | --- | --- |
| Total Tfh (% of CD4^+^ T cells) | 14.03 ± 5.54 | 15.93 ± 6.65 | 0.3888 |
| PD-1^+^ Tfh (% of Tfh) | 43.82 ± 12.71 | 45.50 ± 11.83 | 0.6835 |
| Tfh1 (% of Tfh) | 24.54 ± 10.44 | 17.61 ± 8.30 | 0.0300 |
| Tfh2 (% of Tfh) | 42.20 ± 14.33 | 59.58 ± 21.39 | 0.0199 |
| Tfh17 (% of Tfh) | 26.57 ± 11.70 | 17.05 ± 13.22 | 0.0413 |

Mean ± SD values of circulating Tfh parameters in acute survivors (AS) and acute deceased (AD) among patients without HLH. *p*-values were calculated using two-sided Welch’s t-test for comparisons between non-HLH AS and non-HLH AD groups.

**Supplementary Table S3. Sensitivity analysis of circulating Tfh subsets according to timing of admission-day baseline sampling**

| Parameters | Early admission (≤5 days) (n=34) | Late admission (>5 days) (n=45) | *p* value |
| --- | --- | --- | --- |
| Total Tfh (% of CD4^+^ T cells) | 15.49 ± 6.05 | 15.92 ± 5.99 | 0.761 |
| PD-1⁺ Tfh (% of Tfh) | 46.24 ± 12.71 | 43.09 ± 11.41 | 0.257 |
| Tfh17 (% of Tfh) | 24.12 ± 12.06 | 23.31 ± 13.55 | 0.785 |
| Tfh1 (% of Tfh) | 16.50 [11.90, 29.40] | 20.90 [17.00, 27.20] | 0.122 |
| Tfh2 (% of Tfh) | 50.56 ± 17.39 | 47.46 ± 17.75 | 0.446 |

Values are presented as mean ± SD or median (IQR) as appropriate. *p-*values were calculated using the Mann–Whitney U test or Student’s t-test.
